# Abstracts and Gleanings

**Published:** 1883-09-20

**Authors:** 


### ABSTRACTS AND GLEANINGS.

**Salicylate of Bismuth in Typhoid Fever.**—Prof. Henri Desplats, after a careful study of the antipyretic action of carbolic acid, salicylic acid, salicylate of soda, and resorcine, has demonstrated that these agents have an influence on the temperature and other elements of fever, whatever may be the nature and cause of the fever (typhoid, puerperal, variola, intermittent fever, erysipelas, rheumatism, pneumonia, etc.,); that this action is sure and prompt when these drugs are administered in sufficient quantities; that it is quick, causing rapid elimination. He then studied the accidents which these agents have been said to produce (collapse, convulsions, albuminuria, melanuria, etc.), and has established that they do not cause pulmonary congestion or renal lesions, and may be administered in albuminuria; that, if administered in too large quantity, they may cause collapse, but this collapse is rare and not dangerous; that in very rare cases, when given in enormous doses, they will cause convulsions, but these have never terminated fatally.

He now gives the results of his experiments with salicylate of bismuth in typhoid fever. He has administered it in twenty cases. It is more readily taken than salicylate of soda, being less soluble, and, therefore, of less pronounced taste. It consists of salicylic acid 2 parts, bismuth 1 part, although it keeps more readily if there is an excess of 3–4 parts in the 100 of salicylic acid. It may be given in unleavened bread or slightly aromatic syrup of acacia, in doses of grs. xv-xxx, though he has given from ℈iv-ʒijss in a day. A little seltzer water may be given immediately after it to enable the stomach to retain it better, if that organ be very irritable. It is always advisable that a more or less considerable quantity of liquid be drunk after each dose. The effects obtained are of two kinds, *immediate* and *remote*.

The immediate effects are comparable, when the dose is sufficient, to those produced by carbolic acid, resorcine or salicylate of soda. To observe these, the patient should be closely watched, and the variations of temperature noted. Vulpian's statement that the temperature does not rapidly abate is erroneous, as M. Desplats shows by several cited cases. The immediate effect is never wanting when the dose is sufficient. It produces a less abatement of temperature than carbolic acid, but the sudden rise so often seen after the abatement from carbolic acid, is not observed. Besides the immediate effects, salicylate of bismuth has an incontestable action on the general temperature-curve. As has already been noted by Vulpian, the morning fall of temperature is greater, especially when no salicylate has been administered during the night. Desplats has several times observed that the temperature continued to abate during the forenoon, when the patient had taken no medicine, and thinks it very likely that these late effects are due to absorption of the drug, which is not very soluble. Sometimes, instead of a fall of temperature, he has noticed abundant perspiration coming on during the forenoon, long after the last dose of the salicylate; this seems to confirm the hypothesis of late absorption.

REMOTE EFFECTS.—It is important to know what action the salicylate of bismuth, regularly administered, exercises on the evolution and termination of the disease. Twenty cases are not sufficient from which to draw general conclusions, but they may be of service in drawing conclusions from an additional number of cases. Desplats divides his cases into three groups:

The first includes the cases in which the fever was arrested, in which it may be said that the drug had an abortive action; the second group includes those cases in which the effect was signal, but not so marked; and the third, those cases which were rebellious to treatment, either terminating in death, or seemingly uninfluenced by the drug. Eleven cases are reported in which it had an abortive action. Vulpian has already observed one case in which the fever was arrested on the fifteenth day by salicylate of bismuth. He hesitated to attribute this effect to the drug, as the rose spots were absent. This does not negative the diagnosis of typhoid fever, however, as cases do occur in which the rose spots are absent. Four cases are recorded in which the action of the salicylate was less pronounced; and five cases, all very severe, two recovering, in which there was no apparent effect. In all, twenty cases are recorded. Epistaxis was a rare symptom, and was in no case abundant. Intestinal hemorrhages occurred in two cases, one of which recovered. Delirium was rare, in one case being attributable to the medicine; this was rather subdelirium than true, and there was also deafness. When large doses, ʒij–ʒijss, were given in three or four hours there was, in some cases, a depression of the vital forces, which passed off when the drug was discontinued. The best results were obtained with doses of ℈iv-ʒjs.—*Bulletin Gen. de Therap.*

**Reviving the Still-Born.**—Artificial respiration practiced for an hour at least has so far been considered the most successful means of reviving still-born infants. Prof. Wallace, in his lectures at the Jefferson College, used to mention seveaal cases where, after an hour's trial with artificial respiration, he had at last succeeded in reviving an infant still-born, and where all hope had been given up.

A French physician by the name of Camberdon, has convinced himself, that a very warm bath of a temperature of 50° C. exerted in such cases a far more favorable result than even the method just mentioned. It is a known fact, that infants, who, when born, exhibit a want of vitality, often wonderfully revive when wrapped up in hot blankets or placed into a very warm bath—with one word when replaced into that temperature they had just left in utero. It seems plausible, therefore, that the same procedure may turn out successful in certain cases of the still-born, and we draw, therefore, the attention of our readers to this application of the hot bath.—*Med. and Surg. Rep.*

**Malarial Hæmaturia.**—Dr. J. J. Bland, of Alabama, in Nashville Medical Journal, writes :

On the morning of October 3, 1881, I was summoned to the residence of Capt. J. H. Buford, to see the Rev. Mr. E., who had been indisposed for some time from an attack of malaria, but who had, up to that time, kept up and attended to his ministerial duties. On my arrival, I found the gentleman quite sick. Had a chill about midnight the day before, followed by high fever, and when I saw him (about eight hours after the first rigor) he was suffering from the second. Complained of severe pain in the back, about the lumbar region, aching of lower limbs, shoulders, head, etc. Bowels constipated, tongue coated with a thick, yellow fur, complexion and conjunctiva of a yellow cast. Pulse 149 beats per minute; temperature 104°, and the attendants thought the urine last voided, (which I did not see) too highly colored, and feared it contained blood. Prescribed:

℞ Hydrarg. chlor. mitis.................... } aa grs. v.

Colocynth comp. ext.................... }

M. Et enclose in capsules, No. ij.

Sig. To be taken at once, and followed in four hours, if bowels are not moved, with ℥ss. sulph. magnesia, largely diluted.

℞ Quinia sulph.................................. ʒj.

Ft. Chart, No. vj.

Sig. One powder every two hours.

My parting injunction was for me to be summoned immediately on the next evacuation from the bladder, if the urine contained blood. In about two hours I was dispatched for in haste, and on arriving, I found a copious discharge from the bladder, which was of a very dark color, and contained so much blood, that it had partly coagulated in the vessel. The complexion and conjunctiva, which were of yellow cast at my first visit, had changed in the two hours, to a much deeper hue, and my patient was in a semi-comatose state, and wore a haggard expression. Ordered :

℞ Ergot, fl. ext., (Squibbs')........................ ℥j,

Tr. ferri. chlor.................................. ʒij,

Buchu, fl. ext............................ } aa. ℥ss,

Spts. æth. nit............................ }

Aqua menth. pip., ad........................... ℥jv.

M. Sig. A tablespoonful in water, and repeat every two hours, until the blood in urine diminishes. A warm pediluvium with friction was used, and a mustard plaster to the lumbar region ordered. Other urinary discharges followed, with equally as much blood to the amount voided, as the first evacuation contained, but the discharge was not so copious.

Only a few hours elapsed before there was some appreciable appearance of the urine for the better. But the day passed away, midnight arrived, and the cry was: "The blood still comes." I

was gratified, however, to see that my treatment was having the desired effect, and hence I persevered.

October 4. Patient much improved. Pulse 110; temperature $99\frac{1}{2}°$; urine contains but little blood, and but little pain experienced in voiding it. Bowels well moved during the night of a dark, bilious nature. My friend, Dr. J. M. Hicks, of Goodman, Miss., the patient's family physician and a gentleman ripe in experience in the treatment of this disease, is with me to-day. He approves my treatment, and encourages a perseverance in the same.

October 5. Still improving. Urine diminished in quantity, improved in quality. Takes nourishment in the form of soup and iced sweet milk.

October 7. Patient not doing well. Troubled with epistaxis last night, and the evacuations from the bowels as well as those from the bladder, contained some blood. Slept but little during the night, and is quite nervous to-day. Tenderness over lumbar region more acute, and he is too much nauseated to take food. Ordered the ergot, buchu, etc., mixture, which had been discontinued for two days, to be resumed and kept up until epistaxis, bloody urine and blood in fecal discharges cease:

℞ Potas. bromid.......... } aa ℈ ij

Chloral hydrat.......... }

Syr. aurant. cort.......... } aa ℥ j.

Aqua menth. pip., ad.......... }

M. Sig. A tablespoonful, and repeat every two or three hours, until resting.

℞ Quinia sulph.......... grs. xxxvj.
Enclose in capsules, No. xij.
Sig. One capsule every two hours.

October 8. Slept well last night, and took light nourishment this morning No epistaxis last night, and urine and fecal discharges free from blood this morning. Tongue pale and coated with a white fur. Small quantities of sherry wine in connection with the food was allowed, and a tonic of chloride of iron and the compound tincture of gentian and cinchona was commenced.

October 12. Patient slowly but steadily gaining strength, and is able to sit in an arm chair for an hour or more at a time. I dismiss him with the promise that the tonic will be persevered in until his appetite is good and his strength regained. Since his recovery it has not been my privilege to meet him, but in a recent letter, he writes that he is fleshier than ever before, though not so stout, and still has a weakness and tenderness about his back.

**Cephalhæmatoma in the New-Born.**—Cephalhæmatoma, said the speaker, is a soft, elastic, fluctuating tumor, generally painless, and situated upon one of the cranial bones. It takes place, it seems to me, with somewhat greater frequency than the literature of the subject would lead us to suppose. I have already seen four cases in twelve years' practice. Contrary to the experience of other observers, all four cases have taken place upon the left parietal bones. It is stated by some writers that in the great majority of cases, indeed, in almost all, the tumors have been upon the right parietal bone, inasmuch as it is this bone that is exposed to the pressure of the rigid os uteri in the greatest number of deliveries. This tumor has not, in the cases that have occurred in my practice, made its appearance immediately after birth. Some three or four days have usually elapsed before my attention has been called to the difficulty. It has, in a few cases, been noticed upon both of the parietal bones, although this has not occurred in my practice. When the tumor is first noticed it is a soft and painless enlargement. In the course of a few days a firm bone ridge is usually noticed surrounding the base of the tumor. Th : seat of the difficulty, he said, is between the bone proper and the periosteum, and the enlargement is caused by the rupture of a blood vessel in this position, and the bony ring is simply bony material thrown out from the periosteum to repair this injury. This ridge, or bony ring, does not contract evenly in all directions, and little hard projectious will spring forward from this ridge, showing that the deposits of osseous material does not take place evenly in all directions. As the process of repair goes on, the tumor loses its soft, fluctuating feeling, and in the course of a few weeks nothing can be detected but a slight want of symmetry in the two parietal bones.

The most important question connected with the subject, the speaker said, was its diagnosis, and it appeared to him that there were four difficulties with which it was likely to be confounded, namely: caput succedæneum, congenital encephalolocele or hernia cerebrum, erectile tumors and crania tabes. To show the distinction between these four difficulties and the one under discussion the speaker said the caput succedæneum is an œdematus condition, and it does not fluctuate, being only a difficulty of the scalp, cellular tissues, and blood vessels ; congenital encephalolocele never occurs, with a possible exception, on the cranial bones ; a vascular tumor has sometimes the same boggy feeling noticed in caput succedæneum, but it has no bony ridge ; crania tabes is the soft places found upon the cranial bones in rickety children.

As to the treatment of this difficulty, the speaker said the best method to be pursued was a judicious letting alone, nature, in a great majority of cases, being able to effect a cure without the aid of the medical art.—*Peoria Med. Monthly.*

**Treatment of Gout.**—Dr. N. S. Davis, of Chicago, recommends forty drops of an equal mixture of the acetated tincture of opium and wine of colchicum seeds to control acute paroxysms of gout. This dose may be repeated in an hour if necessary. Oftentimes, one or two doses will abort what threatens to be a very seveae attack· When the paroxysm is under control the same remedies may be repeated in smaller doses, three or four times daily, if any gout remains. We have used this remedy, and can add our endorsement to this distinguished recommendation.—*Med. and Surg. Rep.*

**Examination of Urine.**—Dr. Formad, of Philadelphia, who has recently come into prominence as a microscopist, gives the following succinct rules for the examination of urine:

1. Sediment in the urine has no significance unless deposited within twenty-four hours.

2. Albumen in the urine does not indicate kidney disease unless accompanied by tube casts. The most fatal form of Bright's disease—contracted kidney—has little or no albumen.

3. Every white crystal in urine, regardless of shape, is a phosphite, except the oxalate of lime, which has its own peculiar form—urine alkaline.

4. Every yellow crystal is uric acid, or a urate if the urine is acid, or a urate if the urine is alkaline.

5. Mucous casts, pus, and epithelium signify disease of the bladder (cystitis) or of other parts of the urinary tract, as determined by the variety of epithelium.

6. The urine from females can often be differentiated from the urine of males by finding in it the tesselated epithelium of the vagina.

7. Hyaline casts (narrow), blood, and epithelial casts signify acute catarrhal nephritis. Much albumen.

8. Broad hyaline casts, and epithelial dark granules and oil casts signify chronic catarrhal nephritis. At first, much albumen; later, less.

9. Hyaline and pale granular casts, and little or no albumen, signify interstitial nephritis.

10. Broader casts are worse than narrow casts, as far as diagnosis is concerned, for the former signify a chronic disease.

11. The urine should be fresh for microscopical examination, as the micrococci will change hyaline casts into granular casts, or devour them entirely in a short time.

12. Uric acid in the urine may, in Trommer's test for sugar, form a protoxide of copper, this often deceiving the examiner into the belief that he has discovered sugar. Thus, when urine shows only sugar, other methods of examination should be used—preferentially the lead test.

13. The microscope gives us better ideas of the exact condition of affairs in the examination of the urine than in various chemical tests.—*Southern Clinic.*

**The Parasite of Marsh Fever.**—Dr. Laveran, of the Val-de-Grace, in a communication to the Paris Hospital Society (Union Med., June 12th and 14th), states that the observations which he formerly made in Algeria as to the presence of well-defined parasitic organisms (which he describes at length) in the blood in marsh fevers have been fully confirmed by subsequent researches. Their pathogenic character has been proved by a large number of facts which enable him to come to these conclusions: 1. These parasitic elements exist always in the blood in cases of impaludism; and even when the examination of the blood of the living does not always exhibit them, they are still always to be found, at least after death, in the capillaries of the spleen. 2. They are always found in direct ratio with the severity of the case. In individuals who succumb through some complication in simple intermittent they are found only in small numbers in the liver and spleen; but in those who die from pernicious fever they exist in large numbers in all the organs and vascular tissues.

3. They precede a paroxysm of the disease, and when they are found in the blood we may almost certainly predict that a paroxysm is about to occur, although no elevation of temperature or other morbid sign exists. 4. These elements are never found in diseases unconnected with impaludism. 5. These elements disappear rapidly under treatment by quinine.—*Med. Times and Gazette*, June 23, 1883.

**Ante-Natal Murder.**—In my daily rounds as physician I see grown young men and women in the full enjoyment of life whose mothers appealed to me to destroy them in utero. Oh horrors! what if I had done it, and robbed them of all the experiences of this world? Let every physician and mother in the land, and in the world, understand the enormity of this crime and realize its consequences. A growing evil that has assumed gigantic proportions, is the frequency of ante-natal murder, often out of wedlock, and quite as often in. Do physicians and mothers realize and know that there is no time after conception when there is not life and another soul existent, that shall live on and on through the eternal ages? Do physicians and mothers realize and know that they must meet face to face in spirit life all these ante-natal murdered children, and that they will rise up in judgment against them? Do they know that they will then feel like calling on the mountains and rocks to fall on them and hide them from the objects of their iniquity? Do they know that remorse of conscience will cling to them, and that they will be self-branded as Cain was of old—a murderer? Do physicians and mothers know, that as far as responsibility goes, they might as well take a hatchet and brain the prattling darling of two or three summers as to strangle a soul in uterine life? Then let the physician and mother remember that at conception the decree has gone forth that an immortal soul has been born for eternity, and that they should not stain their hands in human blood.—J. M. WARD, M.D.—*Med. Brief.*

**Cholera Morbus.**—Dr. Cartledge, in the London Medical News, reports a number of severe cases of cholera morbus relieved by injections of ice-water into the rectum, etc., as follows:

In short, my treatment now of the severe forms of the disease is—taken in the stage of evacuations, cramps, etc.—sinapisms to the abdomen, ice by the mouth, and ice-water injections per rectum. The same treatment for the collapse, with possibly some hypodermic stimulation. This is simple medication, but the results can't be bettered in my experience. In milder cases, even in severe, you can use opium with atropia or what not, as suits you. Many of the ordinary cases of sporadic cholera are treated very successfully with the hypodermic administration of morphia and atropia.

In offering an explanation of the action of ice-water injections in this disease, I can only say, it does not seem to be due so much to the local astringent effect of cold as to the strong impression made upon the nerve centers through reflex action by the cold being placed directly in contact with the congested and heated center. It is probable that in severe cases of epidemic cholera a stronger impression might be made and reaction induced by using a rectal tube, thus placing the cold nearer the center of morbid action.

Since writing the above observations, I notice in the Peoria Medical Monthly, where Profs. Pooley and Kinsman, of Columbus, Ohio, have used ice-water injections per rectum in cholera infantum, with good results.—*Louisville Med. News.*

**Ipecac in Labor.**—Dr. Sullivan, in the Nashville Medical Journal, says:

Was called July 9, 1882, to see Mrs. K., in labor with her second child, 6 o'clock p. m. Had been in labor since 6 a. m. Parts rigid with considerable dilatation, pains weak and short with about thirty minutes interval. Gave ipecac, five grains with intention to repeat the dose in half an hour. Before time to give the next dose the pains increased in frequency and force, and in one hour labor terminated.

November 7, 1882. Was called to see Mrs. C., at 1 o'clock a. m. Had been in labor since sundown with her third child. Condition was about the same as the above case. Gave ipecac, 5 grs. In about fifteen minutes she remarked, "Doctor, you ought not to have given me that medicine, it makes these pains harder." And in half hour longer I delivered her of a fine male child to the joy of both parents (it being the first boy).

Mrs. W., in labor with her seventh child. Water discharged at 6 o'clock a. m. Had been in labor all day and night. Called to see her at 1 o'clock a. m. No rigidity about the parts, but with that careless and indifferent mood, which is so often met with in a non-acting uterus, (I use this for the want of a better expression). With the use of one five-grain dose of ipecac the labor was over.

**How to Remove a Tight Ring.**—A novel method of effecting the removal of a ring which has become constricted around a swollen finger, or in any other similar situation, consists simply in enveloping the afflicted member, after the manner of a circular bandage, in a length of flat India rubber braid, such as ladies make use of to keep their hats on the top of their heads. This should be accurately applied—beginning *not* close to the ring, but at the tip of the finger, and leaving no intervals between the successive turns, so as to exert its elastic force gradually and gently upon the tissues underneath. When the binding is completed, the hand should be held aloft in a vertical position, and in a few minutes the swelling will be perceptibly diminished. The braid is then taken off and immediately re-applied in the same manner, when, after another five minutes the finger, if again rapidly uncovered, will be small enough for the ring to be removed with ease.—*Langon, Gaz. des Hop.*

[If the ring be of gold immerse it in quicksilver and an amalgum is formed which is readily broken.—ED REC.]

**Berberis Aquifolium in Syphilis.**—Dr. G. G. Barnabus, in Medical Investigator says: This remedy I regard as one of the most important that we have in our materia medica for the treatment of syphilis; also valuable in scrofula, and in that condition of the blood where boils are the crop.

H. C., a blacksmith, who had been indiscreet in placing his affections in other places than his own home, contracted this disease in the summer of 1879. He tried treatment by the recipe plan, then applied to a physician, who administered to him various compounds with no avail. In January, 1880, being about six months after he had contracted the disease, he applied to me for treatment. His condition now was extremely pitiable, indeed, his tongue was swollen, with great fissures and small ulcers about its edges; the mucus membrane of the mouth and throat was inflamed and excoriated, with here and there deep and eating sores, from which there was a continuous flow of a thick tenacious mucus that caused him to be continually spitting. This condition of his mouth caused him much pain, and gave him much trouble in eating and talking, The anterior perinium was also cracked and sore, from which there was a continuous exudation. I prescribed:

℞ Berberis aquifolium............................ gtts. xv.
Four times per day.

For the excoriations and ulcers, I gave him:

℞ Salicylic acid............................ }
Hydrastis pulv............................ } aa. ʒj.
Triturate and apply two or three times a day.

He commenced to improve immediately, and in the course of two weeks there was a marked change for the better. This treatment was continued until July of the same year, at which time there was not, as far as could be seen, a single symptom of the disease remaining. His wife contracted the same disease from him and applied to me in April for treatment. Her symptoms were not so severe as her husband's, but were the same. I gave her the same treatment, which was continued until August, at which time she was discharged as cured. There has been no return of the symptoms since, it now being three years since they were pronounced cured. I have used this remedy successfully in a number of other cases of this disease as well as scrofula and other kindred maladies.

**A Case.**—Dr. Bowers, Peoria Medical Monthly, reports the following:

On the evening of June 14, 1883, I was called to see a five-year-old daughter of a coal miner; nervo-sanguinous temperament. House low, but on a hill and well drained and airy. The room was very clean and comfortably furnished. Child was lying on the bed in an attitude of natural rest, half-open, expressive eyes. She was hard to arouse and would awaken terrified at objects which she seemed to see in different parts of the room, and immediately drop listlessly back into a state of stupor. Skin intensely hot all over the body.

Pulse, 144; respiration, 25 and arythmic. She said, "I hurt here, and here, and here," meaning all over.

No tumor or tenderness was found in any part of the body. Pupils slightly dilated; cheeks alternately flush and pale. Heart and lungs normal. Tongue centrally coated, red edges and glazed. Urine passed frequently, but plenty. No movement of bowels for twenty-four hours, and then a small dry stool. Vomiting whenever anything was swallowed.

The child showed oppression, but no depression. A history of pertussis and measles, but only slight cough remained, and the child had lately enjoyed good health.

I concluded some ingesta was at the bottom of these pofound symptoms.

To get the bowels open was the thing, but to keep the physic down was the main point.

℞ Hydrag. chlor. mit.......................... 1 grain,
Sodæ bicarb.............................. 30 grains,
Sacch. alb.................................. q. s.

M. Fiat chart, 8. S. One every three-quarter hours.

Aconite tinct. one drop every three-fourths of an hour.

Slight effervescence from stomach on giving the first powder, but no more vomiting. Abatement of the fever with rest until morning. Salts were tried and vomited. John Wyeth & Bros.' comp. (compressed) cathartic pills were given, one at 7 and one at 12. No stools; so the following was ordered—

℞ Fl. ext. sennæ.............................. ½ ounce,

Sodæ sulphas.............................. 1 drachm,

Syr. simp.................................. ½ ounce,

Aq. menth. pip.............................. 1 ounce.

M. S. Two teaspoonfuls every three hours till freely purged.

After a number of doses free catharsis came on with complete abatement of symptoms.

One pill, above mentioned, was vomited, and the other one came away in a stool, both in as good shape as Jonah was.

The stools were composed mostly of young gooseberries and currants.

What I wish to notice is that we might, in children, think there was not much the matter, when there are not very expressive symptoms. while the child is very ill; and again, when there are profound symptoms regard the illness dangerous, when there is only general oppression.

We learned something about piles too.

Hoping some points of interest may be gleaned from these cases, I submit this report.

**Rheumatism Aborted.**—A writer (in Eclectic Medical Journal). claims to arrest rheumatism as follows: "The main points in treatment are, first: Intense fever and high bounding pulse, I commence with verat. vir., 2 drops, aconite, one drop, repeated every hour, till the force and frequency of the circulation are abated, which is sure to be affected in twelve hours if the drugs are good and strong, and are faithfully given. At the same time I combine salicylic acid, 70 grains, and quinine, 20 grains, well rubbed together, then divide into 10 powders, one to be given every two hours.

This treatment has never failed to break the force of the disease in twenty-four hours. The salicylic acid and quinine are continued on the second day at longer intervals to prevent the acute symptoms returning.

"I usually give the quinine and salicylic acid in syrup of wild cherry. If there is much pain, I resort to some anodyne, but avoid opium as much as possible. Cypripedium, or hyoscyamus, is preferred.

**What Means can be Judiciously Used to Shorten the Term and Lessen the Hours of Labor?**—In describing lingering labor, Dr. Jno. Morris, of Maryland, divides it into three stages: First, when the head remains high up; second, when it has descended into the pelvic cavity, but the parts are tense and undilatable; and, third, when the child inhinges with perineum. He explained the procedure to be used in all these conditions, and at what time to employ them. These procedures were: detaching the members around the cervix with the finger in the first stage, dilating with the pulpy part of the finger and stretching it cautiously during each pain. Forcible external compression, pushing the cervix over the occiput; the administration of opium or ergot, but never in first cases, and finally chloroform. These means all failing, the only alternative is the forceps. The doctor further said that if the means he suggested were employed, laceration of the os and perineum, those *betes noir* of modern medical literature, would be obviated, and post-partum hemorrhages, that greatest of all complications in labor, would be prevented.—*Peoria Med. Monthly.*

**Note on Disinfectants.**—Dr. W. E. Buck writes: Most practitioners must have often realized the inefficiency of disinfectants in allaying the fœtor of cancerous ulcers an annoyance which sometimes troubles patients even more than the pain, or the thought of death. I have used the whole round of disinfectants for cancerous ulcers, but all have failed in allaying the fœtor and keeping the ulcer clean. The disinfectants tried were carbolic acid, sanitas, terebene, resorcin, creasote, boroglyceride, chloride of zinc, charcoal, etc. After failure with these, I tried a saturated solution of hyposulphite of soda added to an equal quantity of water, and found it exceedingly efficacious. The ulcerating surface was well syringed and washed with the solution, and was then covered with rags steeped in the solution. The granulations were kept clean, and the fœtor was well kept under. Most disinfectants seem to lose their virtue after a few days' application, but I have used this one for months on the same patient with continuous good effects. It is cleanly, has no smell, does not stain, and is very cheap.—*British Med. Journal.*

**Local Application of Vaseline in Scarlet Fever.**—I have found nothing so efficient in relieving the burning and itching sensation of the eruption of scarlet fever as the inunction of the whole body with vaseline. The vaseline is simply used by being well rubbed upon the surface of the body with the hand, once or twice a day, and continued as long as the patient complains of burning and itching of the skin. These inunctions soothe and calm the patient in an astonishing manner, and are rarely required beyond two or three days.

On the appearance of the stage of desquamation, I have the whole body well sponged once a day for a week with the following wash :

| | | |
|---|---|---|
| ℞ | Hyposulphite of soda........................... | ℥ viij, |
| | Carbolic acid, No. 1,.......................... | ʒ j, |
| | Glycerine...................................... | ʒ jss, |
| | Aqua........................................... | ʒ viij. |

M. S. Shake well, and sponge the body well after the wash has been made tepid by placing the vial containing it in a pan of hot water.

This sponging should be done in a room of equal temperature ; and immediately after each sponging the body should be well dried with a soft towel, and the patient protected against taking cold. This process should be continued for at least a week ; and it has not only the advantage of healing the new skin, but that of disinfecting the particles of desquamated skin, and thereby lessens the infectious character of the period of desquamation.—*Dr. J. B. Johnson in Med. and Surg. Rep.*

**Effects of the Internal Administration of Glycerine.**—Dr. Tisne speaks highly of glycerine as a therapeutic agent internally administered. He states (Gazette des Hopitaux, March 17, 1883), that it causes no irritation to the mucous membrane of the digestive tract beyond exhibiting a slightly increased peristaltic movement. It exerts a beneficial effect upon nutrition, increasing the weight and palliating many of the distressing symptoms in phthisis, such as loss of appetite, diarrhœa, night-sweats, and insomnia. Its action upon the liver is manifested by an increase in size of the organ and by a more abundant flow of bile. It has a diuretic effect and increases the excretion of urea, the chlorides, and the phosphates. The alkalinity of the urine is diminished, and if any pus be present in this fluid it is greatly lessened in amount.—*Peoria Med. Monthly*.

**A New Remedy for Malarial Fever.**—Dr. Carlo Magliere speaks very highly of a remedy which has been in popular use in some parts of this country for some time. It is a decoction of lemons. He had his attention drawn to it while visiting another section of his country, and after experimenting with it was astonished at its beneficial effects in all sorts of malarial fever. He reports some truly remarkable cures effected by it. He recommends the decoction made of the fresh lemon, cut into slices and boiled in a new earthen pot. It is to be given four hours before the fever. He gives the results arrived at with this decoction as follows, and urges further experiments to be made:

1. The decoction of lemons in malarial affections gives results equal to and better than quinine.
2. It is not only active when quinine is active, but even after the latter drug ceases to be active.
3. It is not less active in chronic malarial affections.
4. It does not present any of the disadvantageous effects of quinine.
5. Its administration is possible also in catarrhal conditions of the digestive tract.
6. Its cheapness renders it eminently popular.—*Courier of Medicine*.

**Cannabis Indica in Menorrhagia.**—Two correspondents to the British Medical Journal call attention to the value of cannabis indica in the treatment of menorrhagia. The ordinary tincture may be given in ten or twenty minim doses, repeated once or twice in the twenty-four hours. It has no evident control over hemorrhages from other causes. The following prescription is highly vaunted by Mr. J. Brown, of Bacup, who says that the failures after its use are so few that it may almost be regarded as a specific:

℞ Tinc. cannabis indicæ........................ *m* xxx,

Pulveris tragacanthæ comp.................. ʒ j,

Spts. chloroformi (Br.).................... f. ʒ j,

Aquæ q. s. ad.............................. f. ℥ ij.

M. Of this one ounce is to be given every three hours.—*Ex.*

**Ergot in the Treatment of Congestive Headache.**—Dr. Charles T. Rogers, ef Honolulu, Hawaiian Islands, writes us regarding the above subject, referring to an article by Dr. J. L. Corning [in The Record of December 23d]. Dr. Rogers thinks that the value of ergot in this trouble is not appreciated. He gives it in large doses (ʒ j of fluid extract) and would not be afraid to repeat it within an hour. He combines it, generally, with a full dose of bromide of potassium (gr. xl or more). The combination is much more effective than bromide alone. Dr. R. says that he is not at all afraid to use ergot in large doses. He has seen ℥ ss. given for pulmonary hemorrhage without toxic symptoms following.—*Southern Clinic.*

**Did Syphilis Exist in America Before the Discovery by Columbus.**—In refutation of the affirmative, which has been asserted by some, Dr. William F. Whitney (Boston Medical and Surgical Journal, April 19, 1883) concludes that the evidence presented thus far does not as yet clearly prove the existence of syphilis in this country previous to the landing of the Spaniards. The conclusive proof is still to be furnished by an extensively and symmetrically diseased skeleton, or by a skull presenting a typical case of caries sicca, as described by Virchow, in his Archives, vol. xv, p. 243. The exostosis noted on the bones of the legs of skeletons found in Indian burying mounds and preserved in museums, might have been readily caused by violence and many other agencies than syphilis.

**Pruritus Ani.**—Pruritus ani, says the New York Medical Record, often proves a most annoying and obstinate symptom, persistently refusing to yield to our therapeutic endeavors. It is therefore, very comforting to be assured that we have, in two well known drugs, two equally efficient specifics. Thus, Dr. Steele, of Denver, (Lancet and Clinic), has found quinine sulphate, rubbed up with only sufficient lard to hold it together, a never-failing specific in this affection. He uses it in both pruritus ani and vulvæ. The nearer you get to the full strength of the quinine, the more efficacious will it prove; and some other physician is similarly confident about the local application of Peru balsam. Hence, we are told, there need be no more itching about the anus, and medicine has achieved a new triumph. Selah!—*Gaillard's Med. Journal.*

**Vegetable Nature of Croup.**—Dr. Ephraim Cutter, in a paper read before the American Society of Microscopiats, claims that the false membrane of croup is a parasitic vegetation.

In a report by Prof. P. F. Reinsch, on a specimen furnished him, it is settled that the larynx and trachea bear a remarkable fungoid vegetation, belonging to three, or at least two, different fungi. In the upper part of the larynx are prevalent cells of more rounded form, doubtless different states of evolution belonging to one or two different species of *Hyhomycetes.* The lower part of the larynx, as well as the trachea, was found overgrown with filmentaceous cells, inclosing short, rounded cells, resembling very much the mycelivm, with interspersed spores characteristic of the mucorineæ.—*Ex.*

